# MiRLog and dbmiR: Prioritization and functional annotation tools to study human microRNA sequence variants

**DOI:** 10.1002/humu.24399

**Published:** 2022-05-29

**Authors:** Agnese Giovannetti, Salvatore Daniele Bianco, Alice Traversa, Noemi Panzironi, Alessandro Bruselles, Sara Lazzari, Niccolò Liorni, Marco Tartaglia, Massimo Carella, Antonio Pizzuti, Tommaso Mazza, Viviana Caputo

**Affiliations:** ^1^ Laboratory of Clinical Genomics Fondazione IRCCS Casa Sollievo della Sofferenza San Giovanni Rotondo (FG) Italy; ^2^ Department of Experimental Medicine Sapienza University of Rome Rome Italy; ^3^ Unit of Bioinformatics Fondazione IRCCS Casa Sollievo della Sofferenza San Giovanni Rotondo (FG) Italy; ^4^ Department of Oncology and Molecular Medicine Istituto Superiore di Sanità Rome Italy; ^5^ Genetics and Rare Diseases Research Division Ospedale Pediatrico Bambino Gesù, IRCCS Rome Italy; ^6^ Medical Genetics Unit Fondazione IRCCS Casa Sollievo della Sofferenza San Giovanni Rotondo (FG) Italy

**Keywords:** functional annotation, machine learning, microRNA, noncoding element, single nucleotide variants

## Abstract

The recent identification of noncoding variants with pathogenic effects suggests that these variations could underlie a significant number of undiagnosed cases. Several computational methods have been developed to predict the functional impact of noncoding variants, but they exhibit only partial concordance and are not integrated with functional annotation resources, making the interpretation of these variants still challenging. MicroRNAs (miRNAs) are small noncoding RNA molecules that act as fine regulators of gene expression and play crucial functions in several biological processes, such as cell proliferation and differentiation. An increasing number of studies demonstrate a significant impact of miRNA single nucleotide variants (SNVs) both in Mendelian diseases and complex traits. To predict the functional effect of miRNA SNVs, we implemented a new meta‐predictor, MiRLog, and we integrated it into a comprehensive database, dbmiR, which includes a precompiled list of all possible miRNA allelic SNVs, providing their biological annotations at nucleotide and miRNA levels. MiRLog and dbmiR were used to explore the genetic variability of miRNAs in 15,708 human genomes included in the gnomAD project, finding several ultra‐rare SNVs with a potentially deleterious effect on miRNA biogenesis and function representing putative contributors to human phenotypes.

## Graphical abstract

We developed two tools to analyze and functionally classify miRNA variants.

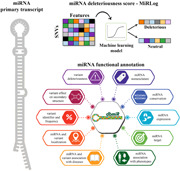

## 1 INTRODUCTION

In the past 20 years, many human genetic variations have been detected thanks to projects aimed at sequencing large datasets using next‐generation sequencing (NGS) approaches. The combined use of whole‐exome sequencing (WES), which focuses on the protein‐coding regions of the genome (about 2%), and computational methods to identify, annotate, and classify protein‐coding variants, has elucidated the molecular bases of several rare genetic diseases and complex traits (Bamshad et al., 2011; Chong et al., 2015). Notwithstanding these achievements, a significant portion (i.e., 50% for Mendelian diseases) still lacks the identification of the genetic cause (Chong et al., 2015).

The recent identification of noncoding variants with pathogenic effects (Spielmann & Mundlos, 2016) or modulating the penetrance of pathogenic protein‐coding variants (Castel et al., 2018), suggests that this class of variations could, at least in part, underlie a proportion of currently undiagnosed cases (Spielmann & Mundlos, 2016). Despite these findings, the functional impact of noncoding variants remains largely uncharacterized. Several computational methods have been developed to address this issue (Nishizaki & Boyle, 2017), exploiting machine learning models built on mixed genomic features, including epigenomic profiles, expression data, and evolutionary measures (L. Liu et al., 2019; Nishizaki & Boyle, 2017). They exhibit only partial concordance (L. Liu et al., 2019) and are not integrated with functional annotation resources, making the interpretation of noncoding variants still challenging. In particular, an integrated system to annotate variants of a specific class of noncoding RNAs, that is, microRNAs (miRNAs), and supporting their classification, is currently lacking.

To date, 1918 human miRNAs have been identified and annotated in miRBase, the miRNA reference database (v22.1; Kozomara et al., 2019). Changes in miRNA expression levels have been extensively studied through several approaches in different cell and tissue types (Mjelle et al., 2019; Nishida et al., 2012; Pérez‐Sánchez et al., 2018), disclosing their role in the pathogenesis of several human phenotypes through the dysregulation of crucial cell pathways (Abdellatif, 2012; Bogucka‐Kocka et al., 2019; Rizzuti et al., 2018). Conversely, miRNA genetic variability and its functional effect are still poorly characterized. An increasing number of studies demonstrate a significant impact of single nucleotide variants (SNVs) on the biogenesis of mature miRNAs and on the strength and specificity of target binding, both in cases of Mendelian diseases, as nonsyndromic hearing loss (Mencía et al., 2009), spondyloepiphyseal dysplasia (Grigelioniene et al., 2019), and in complex traits, as amyotrophic lateral sclerosis (Reichenstein et al., 2019), schizophrenia (Duan et al., 2014), and autism (Williams et al., 2019), among others.

Current knowledge of miRNA sequence variation and its significance is highly affected by the paucity of dedicated tools and the scattered annotation resources. Moreover, the restricted number of established pathogenic miRNA SNVs and small sample sizes of whole‐genome sequencing (WGS) reference cohorts largely limited studies to assess intraspecies human variation in these noncoding regions and its interpretation in terms of pathogenic effect of variants.

To address the issue of interpreting the functional effect of miRNA SNVs, we implemented MiRLog (miRNA Logistic regression), the first scoring approach to support the classification of miRNA variants. MiRLog is a meta‐predictor that integrates multiple scoring systems for noncoding elements, based on a supervised learning approach. We integrated MiRLog in a comprehensive database, dbmiR, which includes a precompiled list of all possible allelic SNVs at each nucleotide position of human miRNAs, and relative annotations based on several data sources, that add biological information at nucleotide and miRNA levels.

We used MiRLog and the functional annotations integrated into dbmiR to explore the genetic variability of miRNAs in human genomes, analyzing WGS data included in the gnomAD project (Karczewski et al., 2020) and we found several ultra‐rare SNVs with a potentially deleterious effect on miRNA biogenesis and function representing putative contributors to human phenotypes.

## 2 METHODS

### 2.1 Noncoding variants scoring systems

We selected tools that predict the deleterious effect of noncoding variants, including eight deleteriousness scoring systems (CADD v1.4, Rentzsch et al., 2019; ReMM 0.3.1, Smedley et al., 2016; Eigen‐PC, Ionita‐Laza et al., 2016; FunSeq 2.1.6, Fu et al., 2014; ncER, Wells et al., 2019; FATHMM‐XF, Rogers et al., 2018; DANN, Quang et al., 2015; LINSIGHT, Huang et al., 2017) and two conservation scores (phyloP, Pollard et al., 2010; phastCons, Siepel et al., 2005), and we scored all the 458,925 possible allelic SNVs occurring in the 152,975 nucleotides of 1869 miRNAs annotated in miRBase v20 (Kozomara et al., 2019). Those methods differ in statistical approaches, training data, and data sources but rely on the assumption that SNVs that are evolutionarily conserved are likely to be deleterious. Since several SNVs (11%) showed missing values from at least one of the scoring systems considered, we imputed missing scores. The imputation was carried out on all the 458,925 possible allelic miRNA SNVs, by an Extremely Randomized Trees (Extra‐Trees) Iterative Imputer (*sklearn* Python library, Pedregosa et al., 2011), composed of 126 base decision trees. We repeated it 10 times using default iterative imputer parameters. We then applied a principal component analysis (PCA) to evaluate the extent of collinearity among the 10 tools.

### 2.2 miRNA variants datasets

To train and test our meta‐predictor MiRLog, we collected two datasets of reference miRNA SNVs (Supporting Information: Tables S1 and S2), “data set 1” and “data set 2.” Data set 1 consists of deleterious and neutral miRNA SNVs. To collect the first class of SNVs, we retrieved variants from HGMD 2020.2 (Stenson et al., 2003) and miRVaS (Cammaerts et al., 2016), selecting SNVs reported as “disease‐causing mutations,” “likely disease‐causing mutations,” “disease‐associated polymorphisms,” and “functional polymorphisms” in HGMD, and functionally validated genetic variants in miRVaS test set. We manually revised the relevant literature to ensure that the deleterious effect was experimentally supported by functional validation assays, retaining only SNVs not reported in gnomAD v2.1 genomes (*N* = 24). As neutral SNVs, we selected miRNA SNVs showing an allele frequency (AF) > 10% (in gnomAD genomes) and neither reported in HGMD 2020.2 nor in miRVaS (*N* = 219). Overall, data set 1 contains 243 SNVs.

Data set 2 is a less stringent data set, consisting of likely deleterious and likely benign miRNA SNVs, not contained in the data set 1. We collected likely deleterious SNVs from HGMD 2020.2 and miRVaS, following the same searching criteria reported for data set 1. In this case, we only considered likely deleterious SNVs already observed (according to gnomAD genomes, *N* = 33). As likely benign miRNA SNVs, we considered SNVs showing an AF <10% in gnomAD genomes, *N* = 10,757), neither reported in HGMD 2020.2 nor in miRVaS. Data set 2 contains 10,790 SNVs.

### 2.3 MiRLog model

We implemented MiRLog, a meta‐predictor based on a supervised machine learning model, to provide deleteriousness scores for all possible miRNA SNVs (except those localizing on the Y chromosome).

MiRLog was built as a classification pipeline. A nested cross‐validation approach was applied to perform two main steps: tuning/training and testing. Both steps were performed on data set 1. As not all the scoring systems considered included scores for SNVs localized on the X chromosome, we added an additional boolean feature that indicated whether the SNV was autosomal or not, to ensure that MiRLog could give different importance to the scoring systems that defined SNVs localized on the X chromosome.

In the tuning/training step, SNVs' scores were firstly standardized through *StandardScaler* (*sklearn* library); then, to reduce the multicollinearity observed among them, SNVs scores were transformed into orthogonal features through a PCA. Next, to add complexity to the model, a quadratic polynomial transformation was applied to features; finally, a bootstrap aggregation (bagging, Breiman, 1996) classifier learned how to discriminate deleterious SNVs starting from the new derived features (i.e., transformed principal components). Both SNVs and derived features were bootstrapped to reduce variance (preventing the overfitting) and ensure that the classifier did not focus only on the most important derived features. Due to the class imbalance of the data set 1, only neutral SNVs were bootstrapped while all the deleterious ones were involved in the training of each base estimator so that each base learner was trained on the same number of SNVs with the same ratio (composition in terms of deleterious/neutral SNVs). As a base estimator, we chose logistic regression, applying L2 regularization to the loss function, to further reduce the possibility of overfitting. We set the number of estimators to 200, as the higher the number of weak learners, the less likely the bagging classifier will overfit.

The overall classification pipeline was implemented in Python, exploiting the *sklearn* and *imblearn* libraries (Lemaître et al., 2017). To tune the L2 regularization power and the bootstrap hyperparameters (deleterious/neutral SNVs ratio and number of derived features randomly extracted), we performed a grid search with a four‐repeated 12‐fold cross‐validation on data set 1, equally distributing the deleterious SNVs in the various folds. Once the best hyperparameters were defined, the same data set (data set 1) was used for the model training. The hyperparameters explored in the grid search process and the best hyperparameters we found for MiRLog are described in Supporting Information: Table S3.

In the testing step of the model performance, we performed 12‐fold cross‐validation on data set 1, and we repeated it four times, with 4 different data set splits. The deleterious SNVs were equally distributed among the folds even in this cross‐validation phase. The model performance was evaluated through a receiver operating characteristic (ROC) curve, and the full performance details are described in Supporting Information: Table S4.

MiRLog approach was then applied on data set 2, to test its predictive performance on an additional data set including a wider spectrum of likely deleterious and likely neutral SNVs. We finally used the MiRLog approach to score all the 458,925 possible allelic miRNA SNVs.

### 2.4 dbmiR database

We developed a manually curated database (dbmiR) that includes all the 458,925 possible allelic SNVs at 152,975 nucleotides in 1869 human miRNAs. As a reference database, we used miRBase v20 (Kozomara et al., 2019), referring to the GRCh37/hg19 assembly, since most of the used databases were built on this assembly. All the functional annotations were retrieved from their repositories (Supporting Information: Table S5), except for Eigen‐PC scores retrieved from regBase (Zhang et al., 2019).

SNVs were annotated following the Human Genome Variation Society (HGVS, den Dunnen et al., 2016) guidelines for variants in noncoding RNAs. miRNA SNVs genomic coordinates were provided for both hg19 and hg38 assemblies (hg38 coordinates were generated through UCSC *liftOver* tool, Hinrichs et al., 2006).

miRNA sequences were retrieved from miRBase and miRNA regions were defined accordingly. miRNA sequences had an extended predicted hairpin precursor, which we defined as “pre‐miRNA,” including a mature region. The mature region was further divided into “seed” (from the first to the eighth nucleotide of the mature sequence), and the “rest of the mature” (from the ninth to the last nucleotide of the mature miRNA) subregions. When a miRNA was annotated in miRBase including two mature miRNAs, we further divided the rest of the pre‐miRNA into a “loop” subregion, between the two mature sequences, and an “out of loop” subregion. This was possible for 923 miRNAs.

Allele frequencies were retrieved from gnomAD v2.1, considering only high‐quality variants, that is, those passing all gnomAD quality filters. Reference variants' identifiers were based on dbSNP152 (Sherry et al., 2001).

SNVs occurring in seeds were annotated using the PolymiRTS database to predict the impact on target binding (Bhattacharya et al., 2014). Information on somatic variations (i.e., tumor, primary tissue, mutation somatic status) was added using COSMIC v89 data set (Tate et al., 2019). SNVs were annotated using the information on associated phenotypes, as reported in ClinVar (version March 2019; Landrum et al., 2018) and based on literature. miRNA disease‐causing SNVs were annotated using data from HGMD 2017.4, retrieved from VEP v100 (McLaren et al., 2016), and manually revised.

SNVs were annotated with multiple scoring systems, including deleteriousness and conservation scores: CADD v1.4, ReMM 0.3.1, Eigen‐PC, FunSeq 2.1.6, ncER, FATHMM‐XF, DANN, LINSIGHT, phyloP, phastCons. We also integrated the score developed in this study, MiRLog. Finally, to evaluate the predicted effect of SNVs on miRNAs secondary structures, miRVaS scores were also computed and integrated.

Official gene symbols were retrieved from the HUGO Gene Nomenclature Committee (HGNC,  Braschi et al., 2019). Genomic localization was reported for each miRNA, that is, exonic, intronic, or intergenic region (based on NCBI RefSeq release 105.20190906; O'Leary et al., 2016). In addition, the predicted localization in a putative cluster was estimated based on the occurrence of another miRNA in 200 nucleotide flanking regions.

miRNAs intolerance to variation was evaluated using the Orion (Gussow et al., 2017) and CDTS (di Iulio et al., 2018) systems. miRNA CDTS scores were reported both for bins of 10 bp (as provided by CDTS system) and also as a mean calculated across each pre‐miRNA. miRNAs expression data were obtained from miRmine (Panwar et al., 2017), containing data on miRNA‐seq in several tissues and cell lines. Transcription factors‐miRNAs regulations were downloaded from TransmiR v2.0 (Tong et al., 2019). Predicted targets were evaluated using TargetScan V7.2 (Agarwal et al., 2015). Experimentally validated targets were retrieved from DIANA‐TarBase V7.0 (Vlachos et al., 2015) and miRTarBase V7.0 (Chou et al., 2018). miRNAs associations with human phenotypes were retrieved from HMDD V3.2 (Huang et al., 2019), PhenomiR 2.0 (Ruepp et al., 2010), and HPO (Köhler et al., 2019) databases. Associations of miRNAs with Mendelian diseases were identified by querying PubMed (Sayers et al., 2021) and a manual revision of selected papers. Information on phenotypes of model organisms was obtained using the Monarch Initiative (McMurry et al., 2016), an integrative database connecting phenotypes to genotypes across species.

### 2.5 miRNA genetic variability

To evaluate miRNA coverage in publicly available data, we analyzed the coverage data of gnomAD v2.1, using the WGS (15,708 cases) and WES (125,748 cases) datasets separately.

Per‐base coverage data corresponding to miRNA sequences was calculated using tabix (from HTSlib 1.9, SamtoolsV; Bonfield et al., 2021) and bedtools *intersect* tools (v2.26; Quinlan & Hall, 2010). Coverage was evaluated as the fraction of miRNA bases covered at a defined depth, calculated as the inverse cumulative relative frequency. We considered a miRNA as “properly covered” if at least 20 reads covered at least 80% of its bases in at least 80% of the sequenced individuals.

miRNA high‐quality SNVs (i.e., those passing all gnomAD quality filters) annotated in the gnomAD genomes were furtherly analyzed, in terms of AF, density, distribution along different regions and nucleotide changes (Transitions/Transversions, Ts/Tv).

We plotted and evaluated miRNA SNVs' AF distribution using the R *ggplot2* package.

We compared SNVs density in miRNA sequences to different genomic regions (i.e., exonic, intronic, and intergenic regions). To this aim, we identified 5,245,679 SNVs in exonic, 79,784,840 in intronic, and 119,764,961 in intergenic regions, from NCBI RefSeq (105.20190906), excluding those overlapping gaps, centromeres, telomeres, and noncoding genes. SNVs density was calculated as the ratio between the number of SNVs in each region and its corresponding length, in kb. SNVs densities were compared using the *χ* ^2^ test, considering as significant a *p* < 0.05.

We analyzed miRNA SNVs density compared to three flanking nonoverlapping, ~100 bp in length, upstream and downstream regions (Saunders et al., 2007). This analysis includes all the 1871 pre‐miRNA regions. SNVs density was also evaluated at the miRNA subregion level, considering miRNAs with two mature miRNAs annotated in miRBase. *χ* ^2^ test was used to compare SNVs densities values (*p* < 0.05). We also analyzed miRNA SNVs distribution along miRNA main regions (i.e., mature miRNAs and the rest of the pre‐miRNAs). A per‐base SNVs density along mature miRNAs was also calculated, as the number of SNVs at each site per 1000 miRNAs (Gong et al., 2012). To evaluate Ts/Tv ratio, VCFtools (Danecek et al., 2011) were used. As genome Ts/Tv ratio, we considered the 204,052,492 high‐quality SNVs detected in gnomAD genomes and localizing outside miRNA sequences. Ts/Tv ratio values were compared using the *χ* ^2^ test, considering as significant a *p* < 0.05.

We analyzed the distribution of MiRLog score for all the 458,925 possible allelic miRNA SNVs at 152,975 nucleotide positions in 1869 miRNAs contained in dbmiR, and for 11,010 miRNA SNVs annotated in gnomAD (genomes), using *ggplot2* package for R. For gnomAD and dbmiR retrieved SNVs, we evaluated MiRLog scores and we compared the distributions using Mann–Whitney test, considering as significant a *p* < 0.05. We also assessed the distribution of MiRLog scores for miRNA subregions for gnomAD and dbmiR SNVs (for the 923 miRNAs with two mature miRNAs annotated in miRBase), and we compared the distributions using the Mann–Whitney test.

We tested the extent of a relationship among miRNAs associated with diseases, their MiRLog average score, and the SNVs density observed. To this aim, we selected gnomAD SNVs localizing in miRNAs associated with at least one disease according to HMDD, calculating, for each miRNA, the number of associated diseases, the average MiRLog predicted score, and the SNVs density. We calculated the Spearman correlation using the *Hmsic* package for R. We also evaluated the extent of a relationship between MiRLog average score and SNVs density observed for miRNAs not associated with diseases, using the Spearman correlation (as described above). We compared distributions of SNVs densities and MiRLog average scores, obtained on both miRNA classes (associated or not associated with phenotypes), using the Mann–Whitney test (considering as significant a *p* < 0.05).

Finally, we evaluated the occurrence of potentially highly deleterious miRNA SNVs in dbmiR. To this aim, we selected miRNA SNVs in the 99th MiRLog percentile (which corresponds to a MiRLog score > 0.98).

## 3 RESULTS

### 3.1 MiRLog: A tool to predict deleteriousness of miRNA SNVs

To assess the deleterious effect of miRNA SNVs, we implemented a meta‐predictor, MiRLog, by integrating 10 scoring systems of noncoding variants, including eight deleteriousness scoring systems and two conservation scores (see Section 2). We firstly scored all the possible allelic miRNA SNVs (*N* = 458,925) at 152,975 nucleotide positions in 1869 miRNAs as annotated in miRBase v20 (Kozomara et al., 2019). To evaluate the extent of redundancy among scoring systems, we performed a PCA, which disclosed that half of the principal components described ~90% of the total variance, thereby revealing nonnegligible multicollinearity among SNVs' prediction scores (Supporting Information: Figure S1).

**Figure 1:**
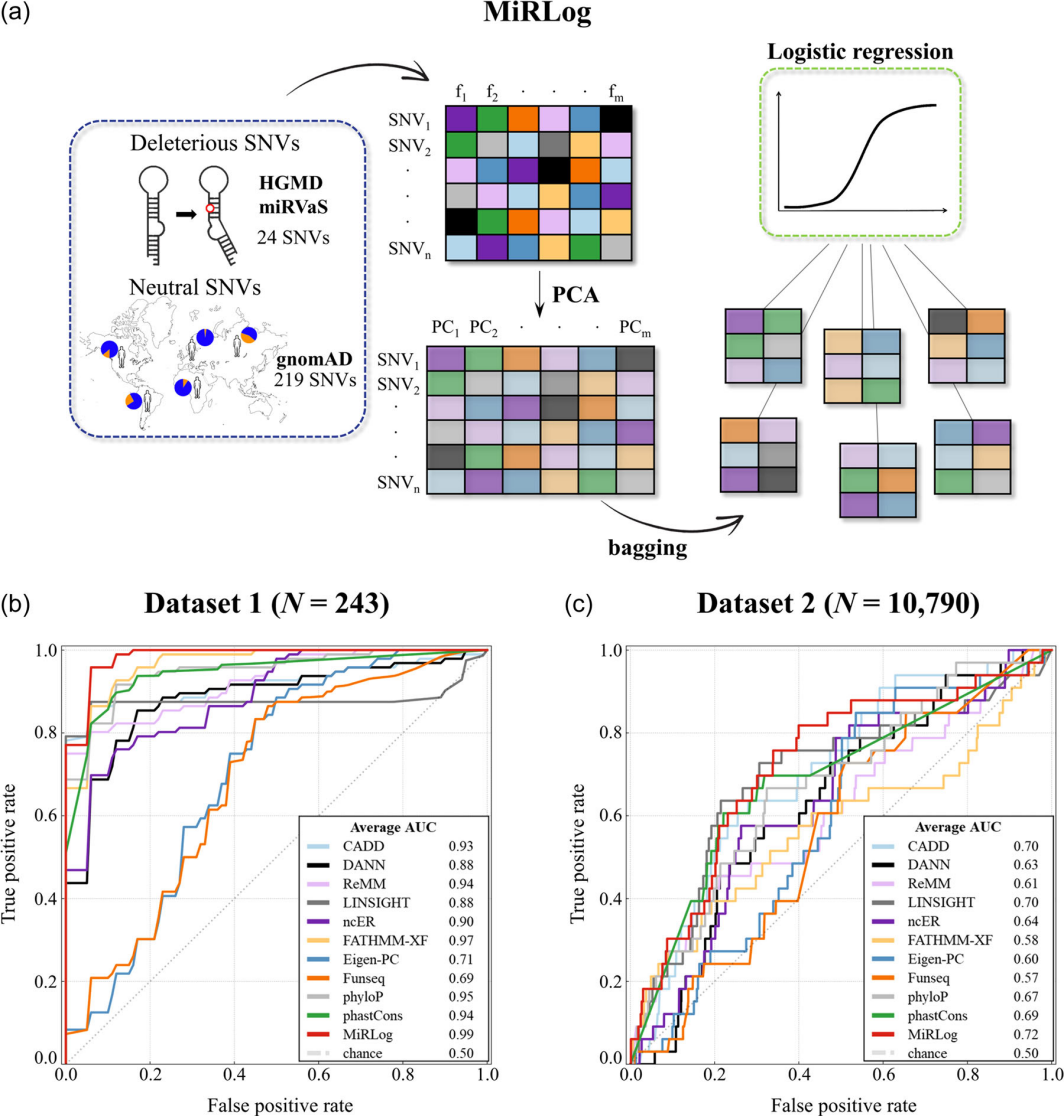
Design and predictive performance of MiRLog. (a) To implement MiRLog meta‐predictor, we collected a data set (“data set 1,” Supporting Information: Table S1) consisting of deleterious and neutral microRNA (miRNA) single nucleotide variants (SNVs) (*N* = 24 and *N* = 219, respectively). SNVs' prediction scores (from *f*_1_ to *f*_m_, upper table) were transformed into principal components (from PC_1_ to PC_m_, lower table) through a principal component analysis (PCA). We used bagging approach to obtain random undersampled datasets, all coming from the “data set 1.” Each generated data set was used to train a logistic regression model. (b) We evaluated MiRLog predictive performance on the “data set 1” (through cross‐validation), (c) and on the “data set 2” (Supporting Information: Table S2), a data set consisting of likely deleterious and likely neutral miRNA SNVs (*N* = 33 and *N* = 10,757, respectively).

To implement our meta‐predictor MiRLog, we firstly collected a “data set 1” composed of deleterious and neutral miRNA SNVs (*N *= 243, Supporting Information: Table S1; Figure 1a). MiRLog is a bagging classifier based on a logistic regression model (Breiman, 1996). MiRLog probabilistic scores range from 0 (neutral SNVs) to 1 (deleterious SNVs), where 0.5 represents the threshold for likely deleterious SNVs.

MiRLog was trained and tested through a cross‐validation approach on “data set 1,” achieving a 0.99 test AUC (Figure 1b), and resulting in the most performant scoring system for miRNA SNVs, followed by FATHMM‐XF (AUC = 0.97). Moreover, we built a second data set (“data set 2,” *N* = 10,790, Supporting Information: Table S2) using more relaxed AF thresholds (see Section 2) and consisting of likely deleterious and likely neutral SNVs, on which we tested MiRLog. Despite the reduction of observed AUC (AUC = 0.72, Figure 1c), MiRLog was found to have better performances than the other single scoring systems on which it is based, also in the case of this data set.

### 3.2 Generation of dbmiR and comparison with other functional annotation tools

**Figure 2:**
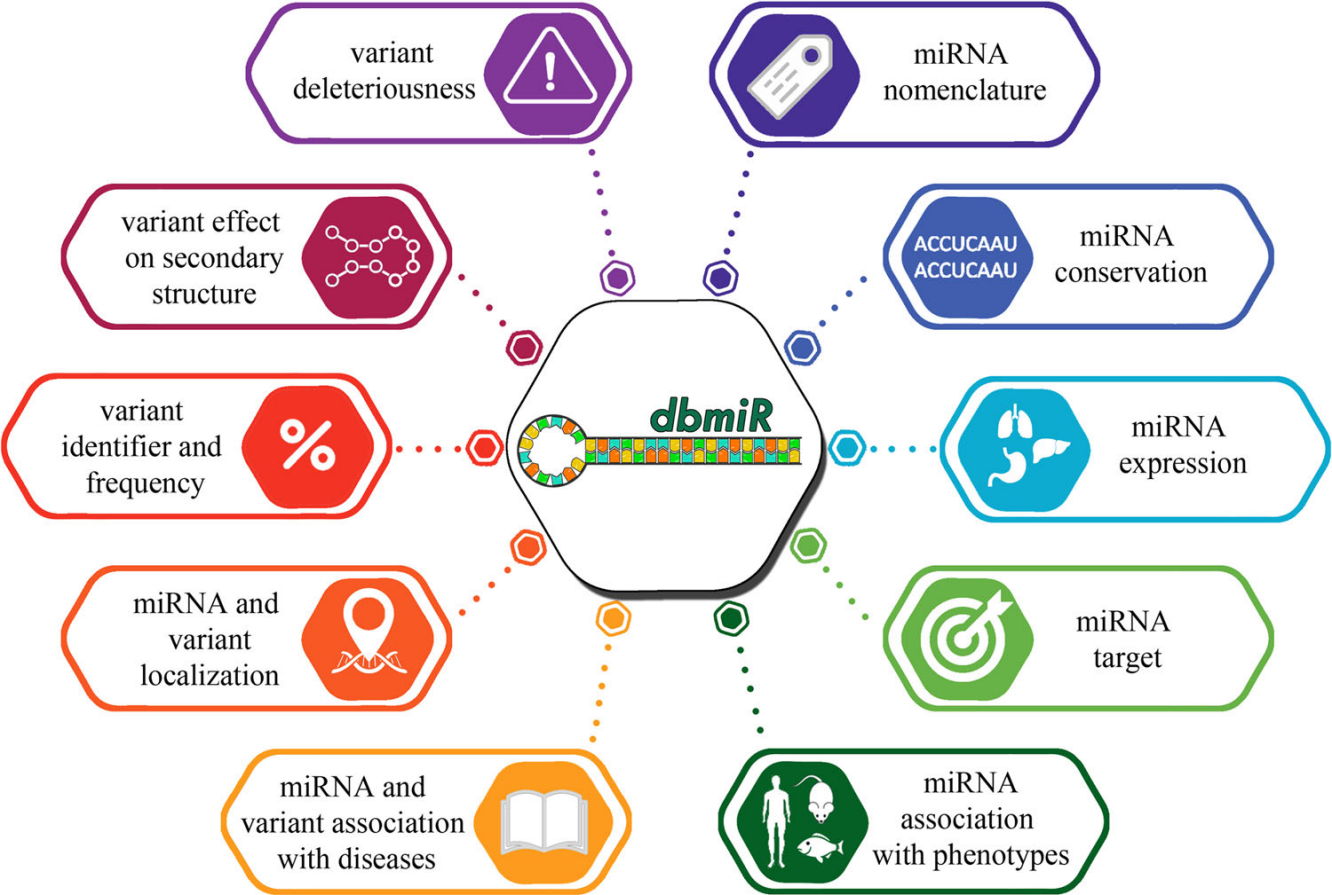
dbmiR database structure. Summary of the functional annotations provided by dbmiR at microRNA (miRNA) and variant levels, classified into categories. For details on resources included in each category, see Supporting Information: Table S5.

We generated dbmiR (Figure 2), an integrated database, providing information on all the 458,925 allelic SNVs at 152,975 nucleotide positions of 1869 miRNAs. Annotations include: SNVs localization in miRNA regions, population AF (gnomAD), variants identifiers (dbSNP), predicted impact on target binding (PolymiRTS), association with   diseases (COSMIC, ClinVar, HGMD, literature), predicted deleterious effect and conservation scores (CADD, DANN, ReMM, ncER, FunSeq2, FATHMM‐XF, Eigen‐PC, LINSIGHT, phastCons, phyloP, and the meta‐predictor implemented in this study, MiRLog), and effect on secondary structure predicted based on the evaluation of the structural impact (miRVaS).

At the miRNA level, dbmiR provides miRNAs genome localization (i.e., exons, introns, intergenic regions, and miRNA clusters), intolerance to variations (Orion, CDTS), tissue/cell expression data (miRmine), interaction with transcription factors (TransmiR), predicted or validated target binding (TargetScan, DIANA‐TarBase, miRTarBase), and the association with human and model organisms' phenotypes (HMDD, PhenomiR, HPO, Monarch Initiative, literature) (Figure 2; Supporting Information: Table S5).

To compare dbmiR to other available databases, we chose those which annotate miRNA variants, adding functional information. To this aim, we analyzed several (~70) available miRNA databases (Tools4miRs database; Lukasik et al., 2016; and literature), selecting specifically those that provide at least one of the following information on SNVs: localization in miRNAs, association with human diseases, effect on miRNAs secondary structure. We retrieved four databases, that is, ADmiRE (Oak et al., 2019), miRNASNP‐v3 (C. J. Liu et al., 2021), MSDD (Yue et al., 2018), and miR2GO (Bhattacharya & Cui, 2015; Table 1). The web‐based platforms, miRNASNP‐v3, MSDD, and miR2GO, allow searching only for miRNA variants already annotated in publicly available databases, such as dbSNP, ClinVar or COSMIC, with miRNASNP‐v3 also providing functional annotation of miRNAs. ADmiRE allows to search also for new miRNA variants but performs a position‐based (and not allele‐specific) functional annotation. This tool provides variants' localization in miRNA sequence motifs and annotates those localized in the proximity of miRNA sequences (up to 100 bp). Differently, dbmiR, not only provides information on both already known and new variants, but also adds annotation based on the specific allele variant. Moreover, unlike the other annotation tools, dbmiR includes several deleteriousness prediction scores, effect on miRNA secondary structure, and association with model organism phenotypes. Finally, it can be easily integrated into a workflow of analysis.

**Table 1:** Comparison of the main features of dbmiR with other microRNA (miRNA) variants' functional annotation tools.

	dbmiR	ADmiRE	miRNASNP‐v3	MSDD	miR2GO
miRNA variants search	▪ Genomic coordinate ▪ dbSNP id	Genomic coordinate	dbSNP id	dbSNP id	dbSNP id
New miRNA variants	Yes	Yes	No	No	No
Localization in a miRNA region	Yes	Yes	Yes	Yes	No
miRNA allelic variants	Yes	No	Yes	Yes	Yes
Functional annotation provided on miRNA variants	▪ Allele frequency ▪ Variants' effect on miRNA secondary structure ▪ Disease‐related variants ▪ Deleteriousness ▪ Conservation	▪ Allele frequency ▪ Conservation	▪ Variants effect on miRNA secondary structure ▪ Variants' effect on targets prediction ▪ Disease‐related variants	Disease‐related variants	Variants effect on targets prediction
Functional annotation provided on miRNAs	▪ Target prediction ▪ Disease associations ▪ Model organisms' phenotypes ▪ Transcription factors ▪ Conservation ▪ Cluster prediction ▪ Expression	▪ Target prediction ▪ Disease associations ▪ Transcription factors	▪ Host gene locus ▪ Biological function ▪ Cluster prediction ▪ Diseases associations ▪ Drug sensitivity ▪ Expression	NA	NA
Availability	Can be implemented in a pipeline	Can be implemented in a pipeline	Web‐based platform	Web‐based platform	Web‐based platform
Year of update	2021	2018	2020	2017	2015

### 3.3 miRNA genetic variability in the human genome

**Figure 3:**
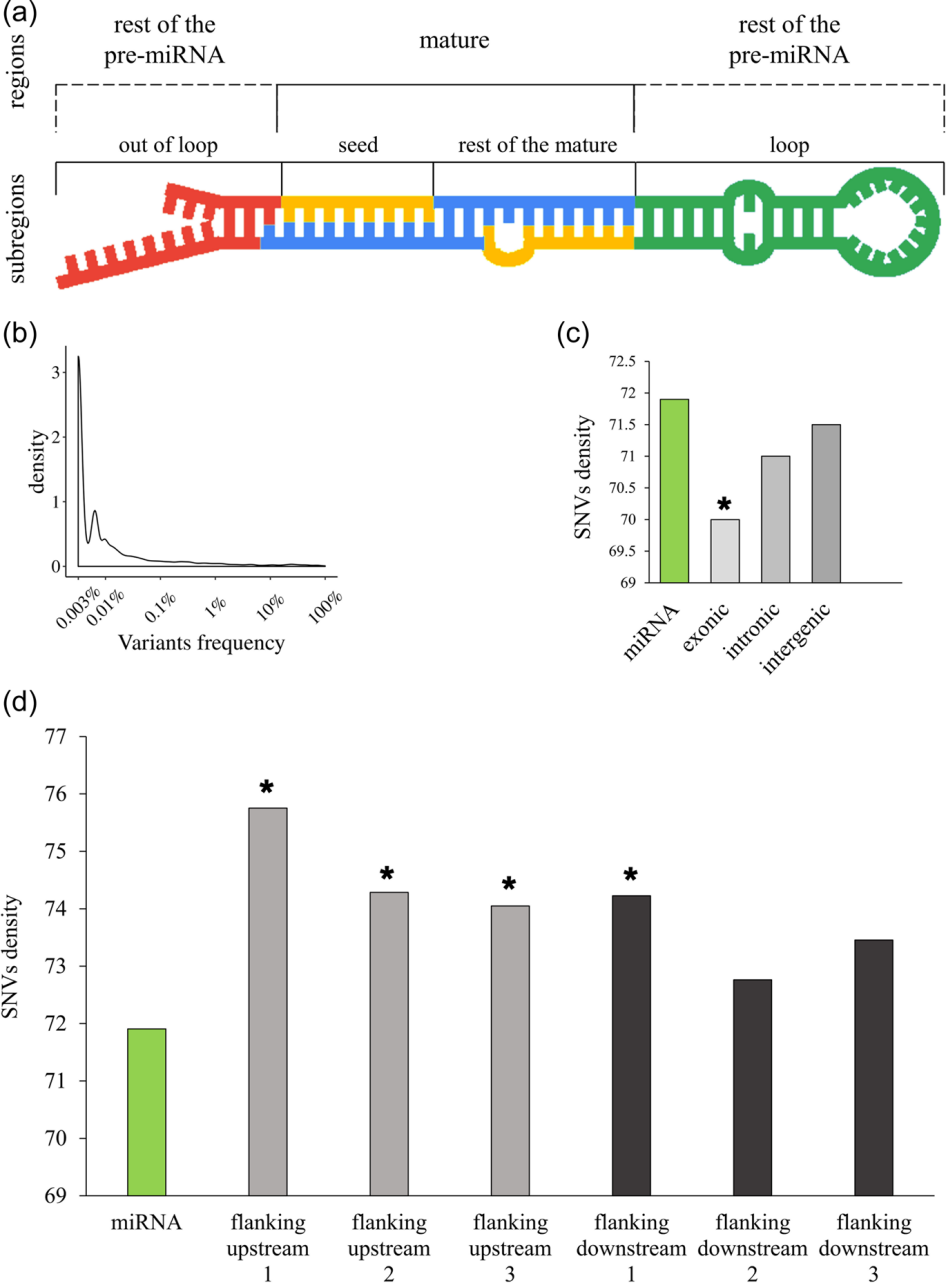
MicroRNA (miRNA) subregions, single nucleotide variant (SNV) allele frequency and density in 15,708 gnomAD genomes. (a) Definition of miRNA subregions. miRNA extended hairpin sequences, as retrieved in miRBase (“pre‐miRNAs”), were divided into mature and rest of the pre‐miRNA regions (i.e., the sequence outside the mature). Mature regions were further divided into “seed” (from the first to the eighth nucleotide of the mature sequence) and the “rest of the mature” (from the ninth to the last nucleotide of the mature miRNA) subregions. The rest of the pre‐miRNA regions were further divided into “loop” (the sequence between the two mature miRNAs), and the “out of loop” subregions. (b) Density distribution of miRNA SNVs allele frequency. (c) SNVs density of miRNAs, exonic, intronic, and intergenic regions. miRNAs had a statistically higher SNVs density compared to exonic regions (indicated with a “*”), and comparable to intronic and intergenic regions. (d) SNVs density distribution in miRNAs compared to three genomic flanking upstream and downstream regions. Flanking regions showing a statistically higher SNVs density than miRNAs are indicated with a “*.” To evaluate statistical differences, we used *χ* ^2^ test (*p* < 0.05).

We evaluated miRNA coverage in publicly available data, that is, gnomAD 2.1. For miRNA sequences' definition, we considered the extended hairpin sequences defined in miRBase (Figure 3a).

miRNAs had an average length of ~80 bp (41 bp min and 180 bp max) in miRBase and mature miRNAs of 22 bp (min 16 bp and max 28 bp). Seeds were 8 bp long. When possible (see Section 2), we further classified the rest of the pre‐miRNA region into a “loop” subregion and “out of loop” subregion. Those subregions had an average length of ~18 bp (4 bp min and 116 bp max) and ~21 bp (1 bp min and 88 bp max), respectively.

A preliminary coverage evaluation of gnomAD data (see Supporting Information: Figure S2) confirmed that the WGS approach could sequence almost all the miRNAs while WES could capture only a fraction (Oak et al., 2019). Overall, gnomAD SNVs represented 2.4% (11,010 variants) of all the possible miRNA SNVs (458,925). The AF values of SNVs localizing in miRNAs (Figure 3b) were mostly (73%) very rare (AF ≤ 0.01%). SNVs density in miRNAs was 71.9 SNVs/kb, higher (*p* < 0.05; Figure 3c) than exonic regions (70 SNVs/kb), and comparable to intronic (71 SNVs/kb) and intergenic (71.5 SNVs/kb) regions, in accordance with previous studies (Telenti et al., 2016).

miRNAs showed a lower SNVs density (71.9 SNVs/kb) compared to flanking regions (75.7 SNVs/kb, 74.3 SNVs/kb, and 74 SNVs/kb for flanking upstream regions 1, 2, 3, and 74.2 SNVs/kb, 72.8 SNVs/kb, and 73.5 SNVs/kb for flanking downstream regions 1, 2, 3, respectively) (Figure 3d), confirming recently reported data (Torruella‐Loran et al., 2016).

At the miRNA subregions level, we did not observe any significant difference in SNVs density (Supporting Information: Figure S3). This result was not influenced by the AF (>5% and >1%) (Supporting Information: Figure S3).

**Figure 4:**
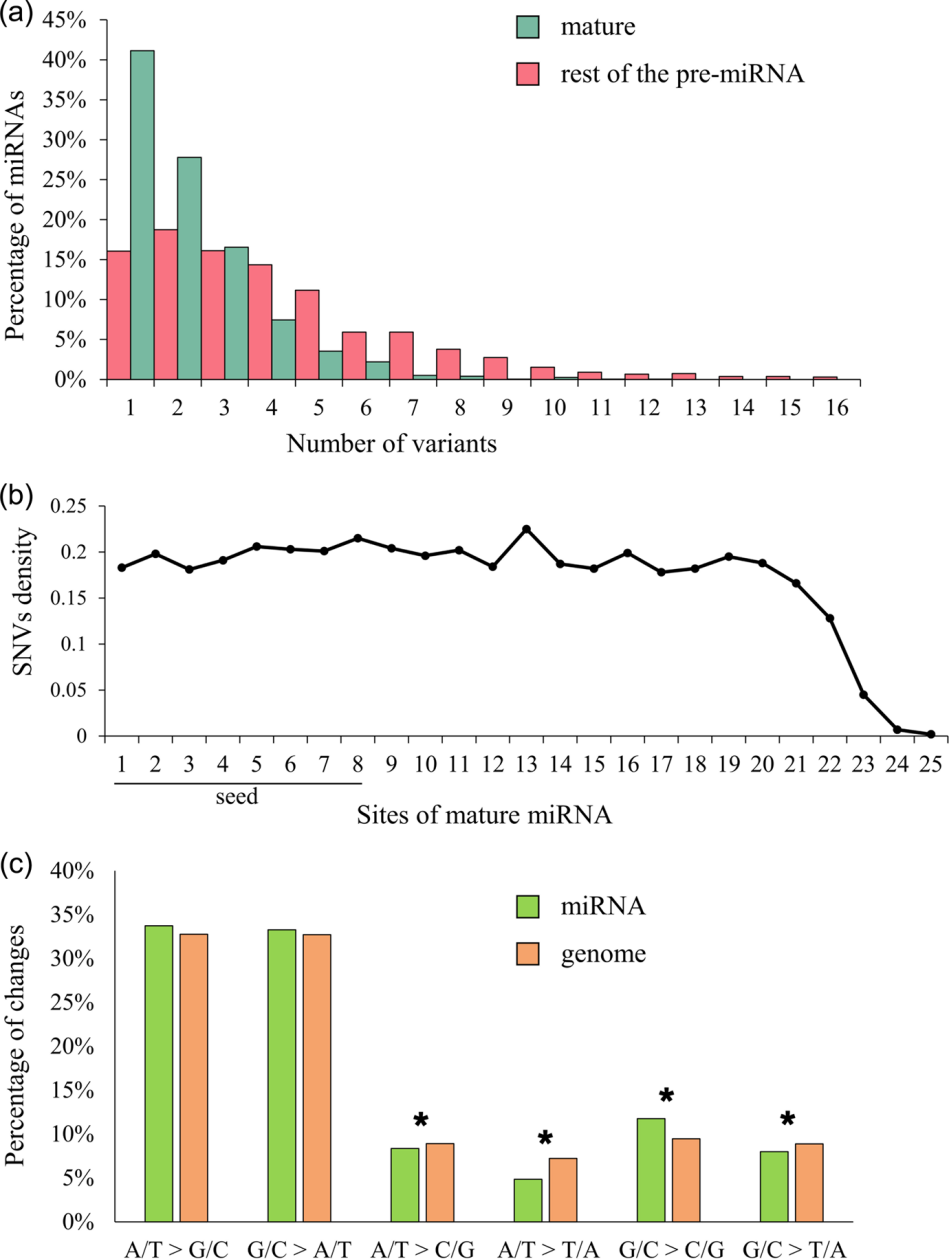
Sequence variability of microRNAs (miRNAs) observed in 15,708 gnomAD genomes. (a) Percentage of mature miRNAs and rest of the pre‐miRNAs showing a defined number of variants. (b) Single nucleotide variant (SNV) density along mature miRNAs. Seed subregion (from the first to the eighth base of mature miRNA) is indicated. (c) Transitions and transversions observed in miRNAs and genome. Transitions were comparable between miRNA and genome. Statistically significant differences in transversions are indicated with a “*.”  For details, see Supporting Information: Table S6. To evaluate statistical differences, we used *χ* ^2^ test (*p* < 0.05).

We then focused on miRNA SNVs distribution in the mature and in the rest of the pre‐miRNA regions (Figure 4a), revealing that 30% (847) of mature miRNAs harbor no SNVs. In mature miRNAs with at least one variant, a median of two SNVs (1–12 SNVs) occurred. Most mature miRNAs (85%, 1664) contained up to three SNVs, while the remaining had up to 12 SNVs. Regarding the rest of the pre‐miRNA, 12% (233) did not have any reported SNV. 6762 variants (61%) occurred in 1638 rest of the pre‐miRNAs, with a median of 3 (1–38), with most of the rest of the pre‐miRNAs (1252, 76%) showing up to five variants. 381 rest of the pre‐miRNAs (~23%) had from 6 to 16 SNVs. The remaining five rest of the pre‐miRNA showed 17, 19, 20, 25, and 38 variants, respectively. The per‐base SNVs distribution along the mature miRNAs (Figure 4b) showed that sites 8 and 13 contained more than 210 variants (215 and 225, respectively), while other sites (1, 3, 12, 14, 15, 17, and 18) showed a reduced variability (less than 190 variants).

Transitions (Ts) in miRNAs (67%; Figure 4c; Supporting Information: Table S6) were much more frequent than transversion (Tv) (33%). Tv values were lower in miRNAs than what observed on average in the genome (35%, *p* < 0.05). Overall, Ts/Tv ratio was higher in miRNAs (2.03) than in the rest of the genome (1.90) (*p* < 0.05). For Ts, miRNA substitutions levels were similar (34% A/T>G/C and 33% G/C>A/T) and comparable to the genome (33% for both changes). Among Tv, miRNAs substitutions G/C>C/G were higher (12%) than those observed in the genome (9%, *p* < 0.05). Differently, G/C>T/A (8% in miRNAs and 9% in the genome), A/T>T/A (5% in miRNAs and 7% in the genome), and A/T>C/G (8% in miRNAs and 9% in the genome) levels were lower in miRNAs than in the rest of the genome (*p* < 0.05).

**Figure 5:**
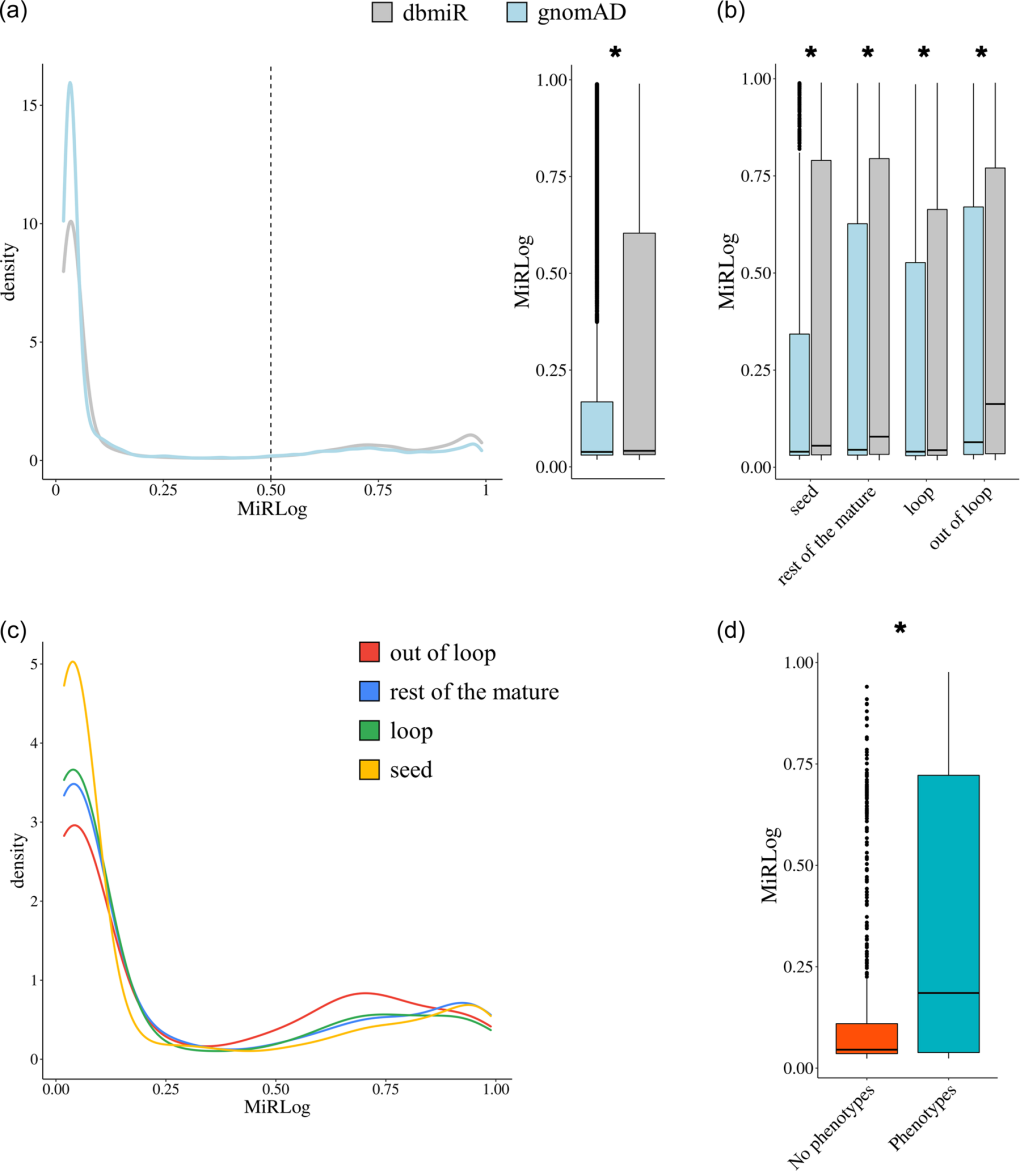
MiRLog score distribution analyses. (a) MiRLog score distributions for microRNA (miRNA) single nucleotide variants (SNVs) in gnomAD and in dbmiR. (b) MiRLog score distributions for miRNA subregions for gnomAD and dbmiR SNVs, localizing in miRNA with two mature miRNAs annotated in miRBase. (c) MiRLog score density distribution for gnomAD SNVs (localizing in miRNA with two mature miRNAs annotated in miRBase) at subregions level. (d) MiRLog score distributions comparison between miRNAs associated with at least one phenotype (light blue) and those not yet reported in association with a disease (according to HMDD, orange). To evaluate statistical differences, we used Mann–Whitney test (*p* < 0.05).

We analyzed the MiRLog score distribution (Figure 5a) of all the 458,925 SNVs at 152,975 nucleotide positions of 1869 miRNAs annotated in dbmiR disclosing that 125,667 SNVs had a MiRLog score > 0.5 (73rd percentile, Figure 5a). For gnomAD SNVs, we observed that 2235 variants had a score >0.5 (80th percentile). The occurrence of potentially highly deleterious SNVs in the reference gnomAD cohort cannot be ruled out as this data set is not enriched for individuals with severe pediatric disorders, but individuals with severe, eventually adult‐onset diseases, may be included (Karczewski et al., 2020).

The average MiRLog score of observed SNVs (i.e., gnomAD) was lower than the predicted SNVs' (i.e., dbmiR) average score (0.20 versus 0.26, respectively; Figure 5a). The distribution profile of MiRLog scores for all the possible SNVs was consistently higher (Mann–Whitney, *p* < 0.05) compared to the distribution of observed SNVs' scores, with observed SNVs showing a greater proportion of nondeleterious variants (MiRLog scores closer to 0) and, conversely, fewer highly deleterious SNVs than expected (Figure 5a). The same results were obtained at subregion levels (Figure 5b, Mann–Whitney, *p* < 0.05). Moreover, we found that the distribution of MiRLog scores of observed SNVs was higher in the out of loop subregions than those in the other ones (Mann–Whitney, *p* < 0.05; Figure 5c).

Then, we considered miRNAs associated with at least one disease (according to HMDD), for which at least one SNV has been reported in gnomAD. For these 908 miRNAs (Supporting Information: Table S7), we evaluated the extent of collinearity among the observed SNVs density, the number of miRNAs' associated diseases, and their average deleteriousness MiRLog score (Supporting Information: Figure S4a, see Section 2). We observed that the SNVs density and miRNAs' associated phenotypes showed an anti‐correlation (Spearman correlation = −0.20, *p* < 0.05, Supporting Information: Figure S4a), confirming previous results on smaller cohorts (Han & Zheng, 2013). Moreover, miRNAs average MiRLog scores were negatively correlated to the observed SNVs density (Spearman correlation = −0.27, *p* < 0.05, Supporting Information: Figure S4a,b), while they were positively correlated to the number of associated diseases (Spearman correlation = 0.61, *p* < 0.05, Supporting Information: Figure S4a). Overall, these findings suggested that the higher the number of diseases a miRNA is associated with, the lower the SNVs density it tends to accumulate, and the higher the deleterious effect the SNVs exhibit.

Moreover, we compared results obtained on miRNAs associated with phenotypes to those not yet associated (i.e., 890). We found that, although miRNAs not associated showed a significantly higher SNVs density compared to miRNAs associated with phenotypes (Supporting Information: Figure S4c), they showed significantly lower MiRLog average scores (Figure 5d), not correlated in any direction with SNVs density (Supporting Information: Figure S4d).

Finally, we evaluated miRNA SNVs predicted to have a high deleterious effect according to MiRLog (>0.98). Of the 4590 SNVs identified in dbmiR (Supporting Information: Table S8), 39 were ultra‐rare (AF < 0.01%) and 4540 not annotated in gnomAD, with 24% of them localizing in 22 miRNAs, for which at least one abnormal phenotype has been observed in model organisms (i.e., Monarch Initiative), suggesting them as candidate miRNAs contributors to human phenotypes.

## 4 DISCUSSION

In this study, we implemented a functional scoring tool, MiRLog, a supervised learning approach to prioritize miRNA SNVs with a potentially deleterious effect, and dbmiR, a database to functionally annotate miRNAs, two resources that could support the interpretation and classification process of miRNA variations.

Recently, the accurate prediction, prioritization, and classification of the noncoding variants' effect on the regulatory architecture of the human genome have emerged as crucial issues. Several methods have been developed to address them, primarily based on functional annotations and cross‐species conservation. Those approaches predict the functional effect of variants localizing in different regulatory regions, and they are usually trained on datasets consisting of a relevant number of noncoding mutations. However, those algorithms predict the functional effect of heterogeneous noncoding elements (i.e., promoter, enhancer, noncoding RNAs, etc.), affecting different molecular mechanisms at transcriptional and posttranscriptional levels, without considering any specific class of noncoding elements. Moreover, the many approaches to evaluating noncoding variants often result in discordant predictions that are difficult to integrate and reconcile (L. Liu et al., 2019).

Regarding functional annotation, currently available tools to localize and annotate miRNA SNVs (e.g., VEP, ANNOVAR, SnpEff) usually fail in this task, due to the lack of a reference sequence database or to the misinterpretation of the functional effect compared to protein‐coding genes (Oak et al., 2019). Then, once localized in a miRNA, biological annotation at variant and miRNA levels is affected by a limited number of available annotation tools and the scattered annotation resources.

To address these issues, we developed dbmiR, a database providing information on all the possible allelic SNVs at each nucleotide position of 1869 miRNAs, which represents a comprehensive resource of biological miRNA‐related knowledge, that can be integrated into a workflow of NGS data analysis. dbmiR integrates data on population AF, impact on target binding, association with diseases, predicted deleterious effect, conservation scores, and effect on secondary structure. At the miRNA level, several biological annotations have been included, such as intolerance to variations, tissue/cell expression data, target binding, and association with human and model organisms' phenotypes. Furthermore, to predict the potential deleterious effect of miRNA SNVs, we implemented MiRLog, a miRNA‐specific meta‐predictor. The major limitation of our approach is represented by the small number of deleterious SNVs included in the data set used to train and test MiRLog. To date, only a limited number of confirmed deleterious miRNA variants have been reported, which can be the consequence of a strong bias due to the historical focus on protein‐coding variations and the challenge to understand and interpret a variant in a noncoding region.

For this reason, MiRLog could not quite effectively generalize its predictive performances, when applied to a wider spectrum of likely deleterious and likely neutral SNVs. Moreover, as MiRLog is a meta‐predictor, the AUC reduction is in line with the reduction of the AUCs of the scoring systems on which our approach is based. We expect that the use of MiRLog could support the identification of new potentially deleterious miRNA SNVs that, once validated and functionally tested, could be used to integrate the datasets and, therefore, increase our approach's performances.

We used the resources developed in this study to explore human miRNA variability, through the analysis of one of the broadest, to our knowledge, cohorts of human subjects (i.e., gnomAD genomes cohort). The ratio between transitions versus transversions was lower than previously reported (Wang et al., 2015), maybe due to the higher number of analyzed variants. Interestingly, human miRNA transversions were statistically less represented than those observed in the rest of the genome. This result could reflect a specific functional role for this type of nucleotide substitution that can introduce DNA structure alterations, transcription factor binding disruptions, and changes in regulatory elements' activity (Guo et al., 2017).

SNVs were mostly ultra‐rare and their average density was slightly higher than previously observed (Telenti et al., 2016). miRNA density was lower than those of flanking regions, confirming reported data on smaller cohorts (Gong et al., 2012; Saunders et al., 2007; Torruella‐Loran et al., 2016).

We did not find any statistical difference in SNV density in miRNA subregions, that is, seed, mature, loop, and out of loop subregions, even if a trend could be observed, with fewer variants in seed compared to mature, and fewer in mature compared to the rest of the pre‐miRNAs. The AF did not influence this result, suggesting that variants in different regions of hairpin precursor could equally affect miRNA function, likely through the perturbation of biogenesis and targeting.

We found that observed variants were fewer than predicted and with a lower deleteriousness score, suggesting that variations that could exert a functional effect are less represented in control subjects. Interestingly, this applies to all subregions of miRNA, that is, seed, rest of the mature, loop, and out of loop. For the latter region, we observed a higher MiRLog score on average than the other regions, suggesting that it may accumulate more variants with mildly deleterious effects and is, therefore, more tolerant to variations with a lower impact on miRNA biogenesis and targeting.

Our observation that miRNAs associated with diseases tend to accumulate SNVs, which generally have a higher deleterious effect, is in accordance with this hypothesis and was demonstrated in several cases of both monogenic (Grigelioniene et al., 2019; Mencía et al., 2009) and complex traits (Duan et al., 2014; Reichenstein et al., 2019; Williams et al., 2019).

In conclusion, we developed a functional scoring tool, MiRLog, and a database, dbmiR, to perform functional annotation of miRNAs. dbmiR, which can be integrated into a workflow of NGS data analysis, potentially maximizes the power of biological annotations, showing an increased efficacy to accurately characterize miRNA variations at a base‐wise resolution. Our composite strategy significantly improved the prediction accuracy that could provide relevant insight into disease mechanisms, underlying both Mendelian and complex traits, and allowed us to further suggest that SNVs in miRNA sequences likely affect their regulatory function, potentially underlying pathogenic mechanisms of human diseases.

## WEB RESOURCES

miRBase: https://www.mirbase.org/

gnomAD: https://gnomad.broadinstitute.org/

HGMD: http://www.hgmd.cf.ac.uk/

Tools4miRs: https://tools4mirs.org/

## CONFLICTS OF INTEREST

The authors declare no conflicts of interest.

## Supporting information

Supporting information.

Supporting information.

Supporting information.

Supporting information.

Supporting information.

## Data Availability

dbmiR tool is freely available for download at https://doi.org/10.5281/zenodo.6498717. It can be used only for noncommercial purposes because of the licenses associated with some of the included tools and databases.
