# Supplementary material for: MiRLog and dbmiR: Prioritization and functional annotation tools to study human microRNA sequence variants
Source: Hum Mutat. 2022 May 29;43(9):1201–15. doi: 10.1002/humu.24399 (PMC9546175; doi:10.1002/humu.24399)
Supplement: Supplementary file 5 — Supporting information. [file HUMU-43-1201-s005.pdf]

Supplementary Material

MiRLog and dbmiR: prioritization and functional annotation tools to study human microRNA sequence variants

Agnese Giovannetti[1], Salvatore Daniele Bianco[2,3], Alice Traversa[1], Noemi Panzironi[1], Alessandro Bruselles[4], Sara Lazzari[2], Niccolò Liorni[2,3], Marco Tartaglia[5], Massimo Carella[6], Antonio Pizzuti[2], Tommaso Mazza[3], Viviana Caputo[2]

1. Laboratory of Clinical Genomics, Fondazione IRCCS Casa Sollievo della Sofferenza, San Giovanni Rotondo (FG), Italy.
2. Department of Experimental Medicine, Sapienza University of Rome, Rome, Italy.
3. Unit of Bioinformatics, Fondazione IRCCS Casa Sollievo della Sofferenza, San Giovanni Rotondo (FG), Italy.
4. Department of Oncology and Molecular Medicine, Istituto Superiore di Sanità, Rome, Italy.
5. Genetics and Rare Diseases Research Division, Ospedale Pediatrico Bambino Gesù, IRCCS, Rome, Italy.
6. Medical Genetics Unit, Fondazione IRCCS Casa Sollievo della Sofferenza, San Giovanni Rotondo (FG), Italy.

Corresponding author: Viviana Caputo
Department of Experimental Medicine, Sapienza University of Rome, Viale Regina Elena, 324, 00161 Rome, Italy
e-mail: viviana.caputo@uniroma1.it

Table of contents

Supp. Figure S1. 3
Supp. Figure S2. 5
Supp. Figure S3. 7
Supp. Figure S4. 9
Supp. Table S3. 10
Supp. Table S4. 11
Supp. Table S5. 12
Supp. Table S6. 15

Supp. Figure S1.

Principal Component Analysis (PCA). (a) PCA of the ten scoring systems used to annotate all the possible allelic miRNA SNVs (N = 458,925). (b) PC1 vs PC2 analysis performed on SNVs belonging to dataset 1 (Supp. Table S1). (c) PC1 vs PC2 analysis performed on SNVs belonging to dataset 1 and dataset 2 (Supp. Tables S1, S2).

a)

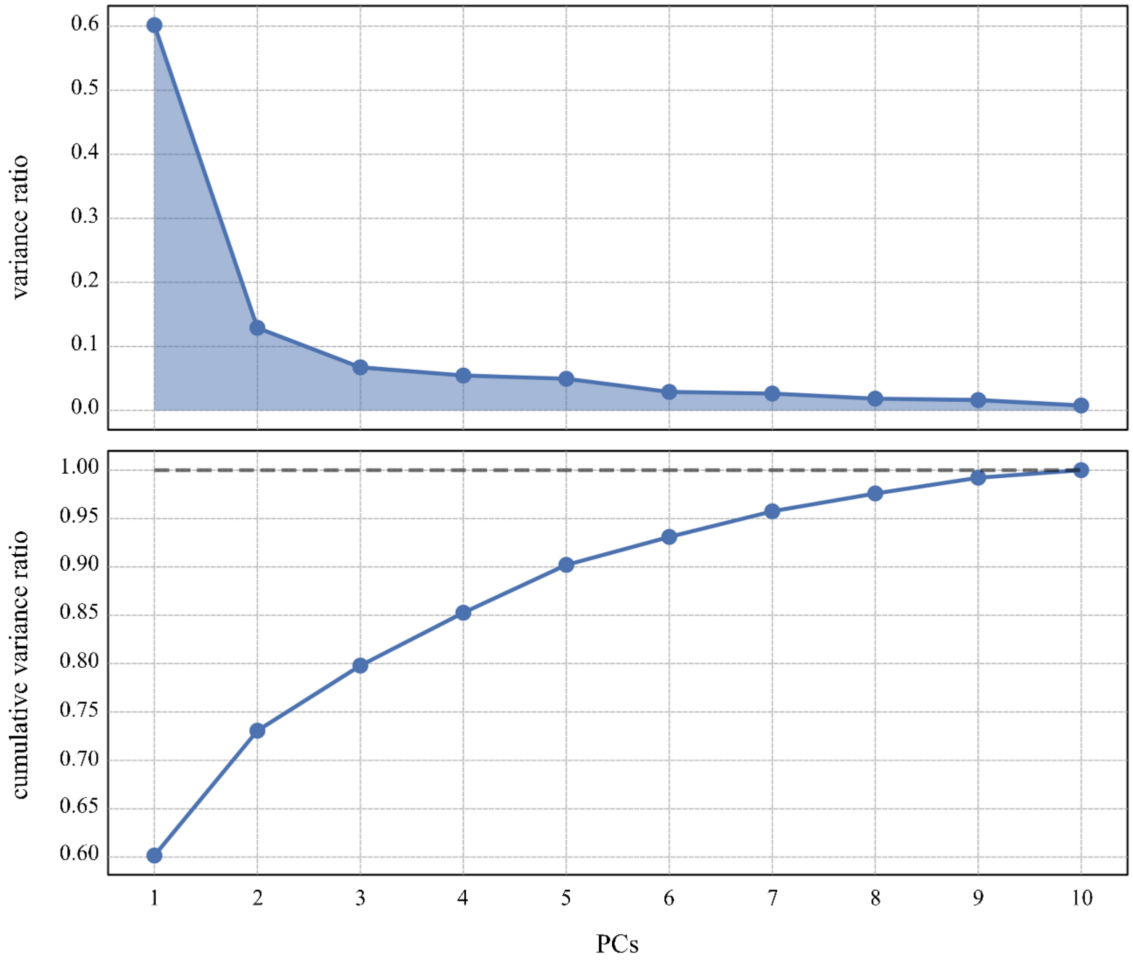

b)

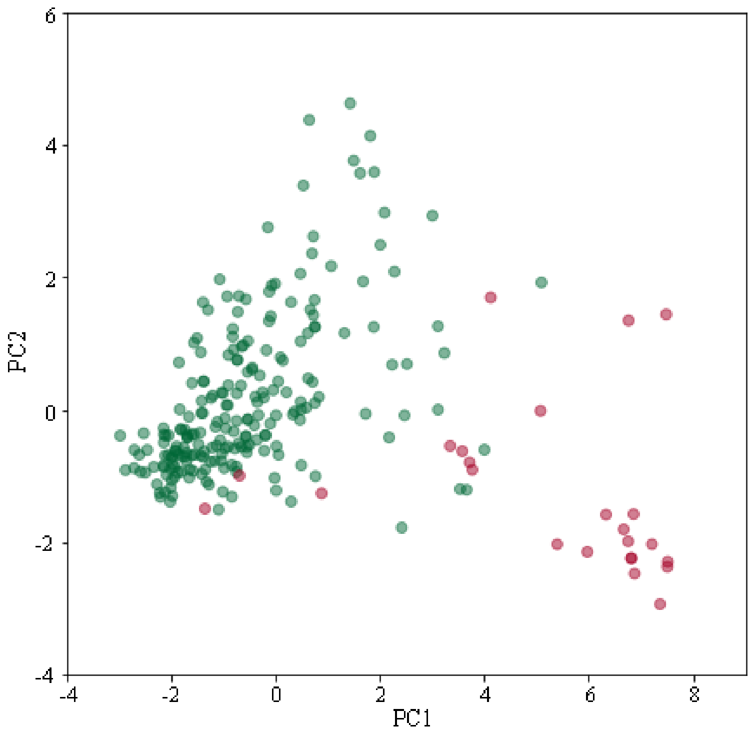

c)

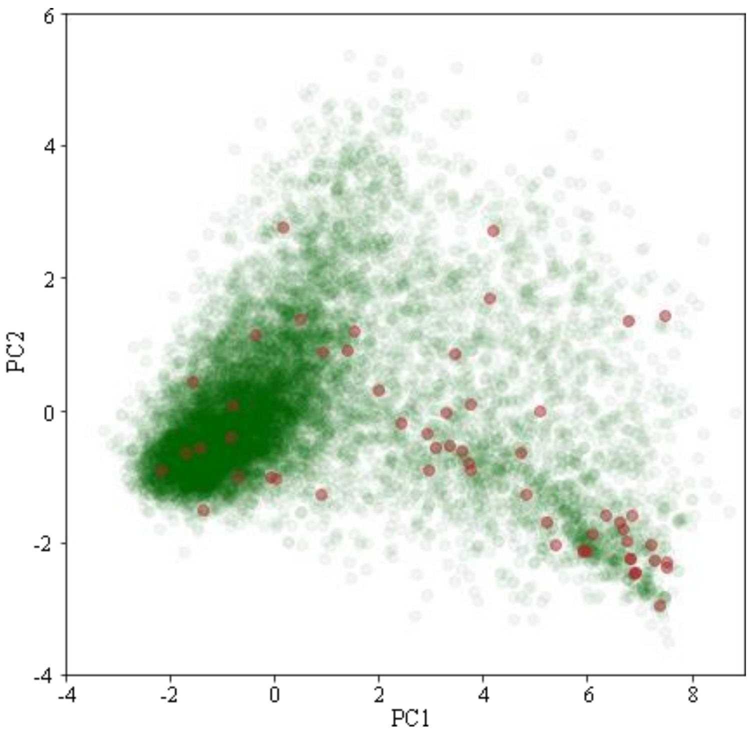

Supp. Figure S2.

MiRNA coverage in gnomAD 2.1 publicly available data. (a) We evaluated coverage of miRNAs sequences in gnomAD WES (dashed line) and WGS (continuous line) data. The graph shows the cumulative relative frequency of miRNA sequences (measured in bp) captured at a defined depth. (b) We evaluated the number of miRNAs captured by WES and WGS approaches. Each cell shows the number of miRNAs with the corresponding percentage of properly covered bases in WES (rows) and in WGS (columns). We considered a miRNA as "properly covered" if at least 80% of its bases resulted covered by at least 20 reads in at least 80% of the sequenced individuals.

Analysis of WGS data showed that 94% of miRNA bases were covered by at least 20 reads and that 79% of miRNAs (1473) can be considered as "properly covered". Differently, coverage of miRNA bases of WES data was 46% at a depth of at least 20X with 22% (412) of miRNAs resulting as "properly covered".

a)

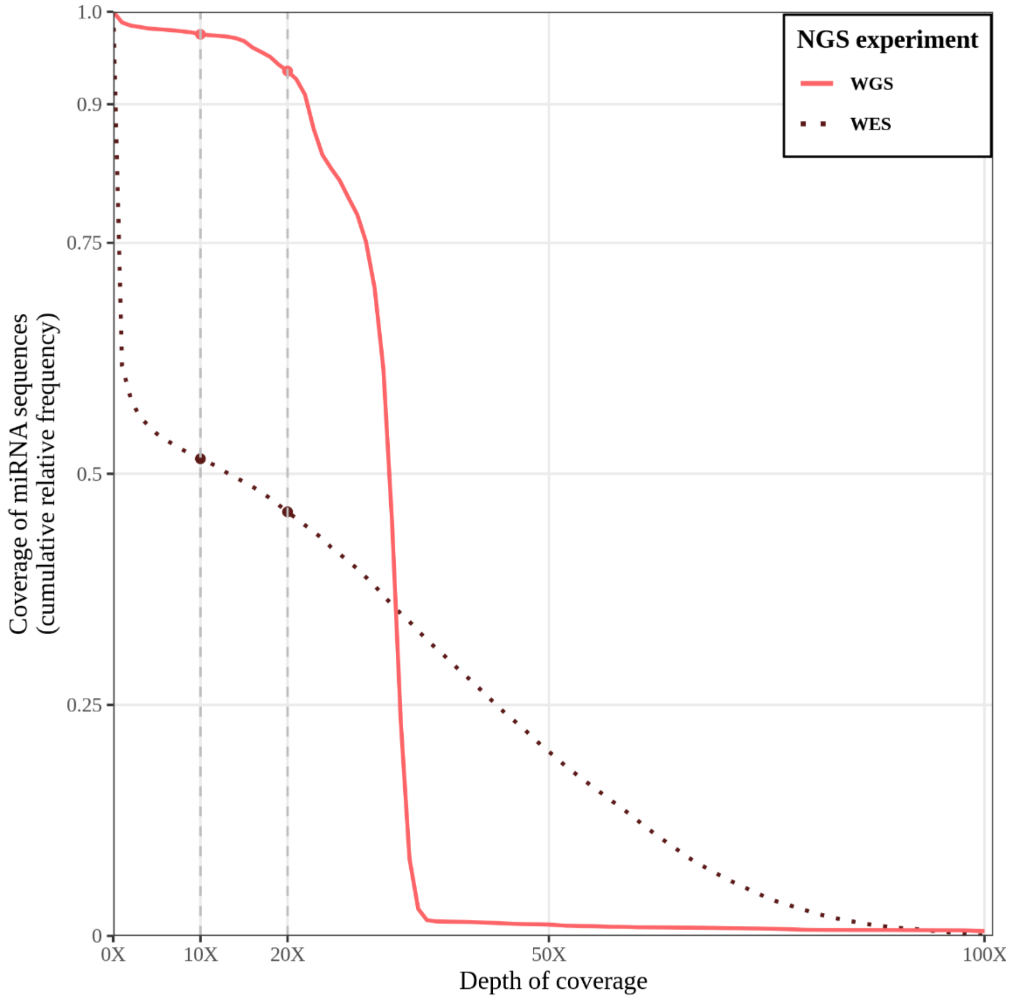

b)

WES

≥80%	24	0	1	1	386
60%-80%	8	1	0	0	49
40%-60%	6	0	0	0	53
20%-40%	7	0	0	0	28
<20%	314	11	14	11	957
	<20%	20%-40%	40%-60%	60%-80%	≥80%

WGS

Supp. Figure S3.

SNVs density in miRNAs with two mature miRNAs annotated in miRBase. SNVs density distribution in miRNA subregions compared to three, ~100 bp in length, genomic flanking upstream and downstream regions (1, 2, 3, respectively), for all variants (a), those showing an AF > 5% (b) and an AF > 1% (c). Subregions showing a statistically higher SNVs density are indicated with a coloured "*", based on the colour of miRNA subregion to which they refer. To evaluate statistical differences, we used Chi square test ($p < 0.05$).

a) **All variants**

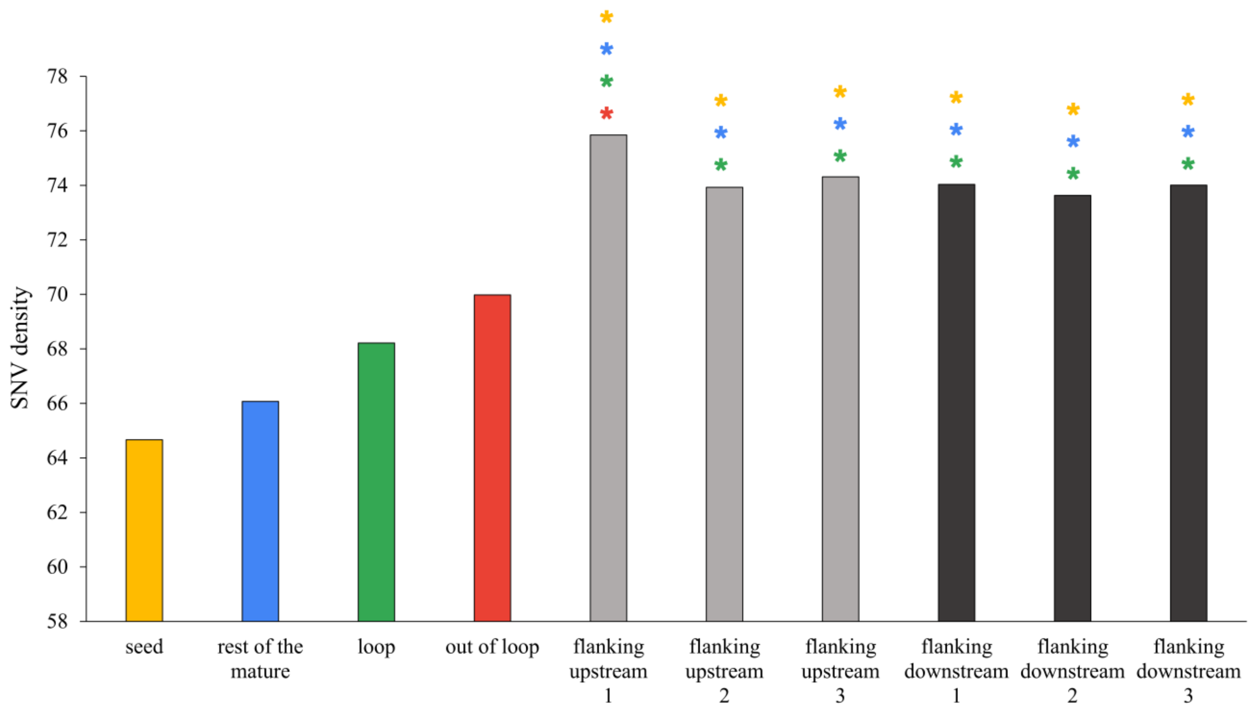

b) **Variants with AF > 5%**

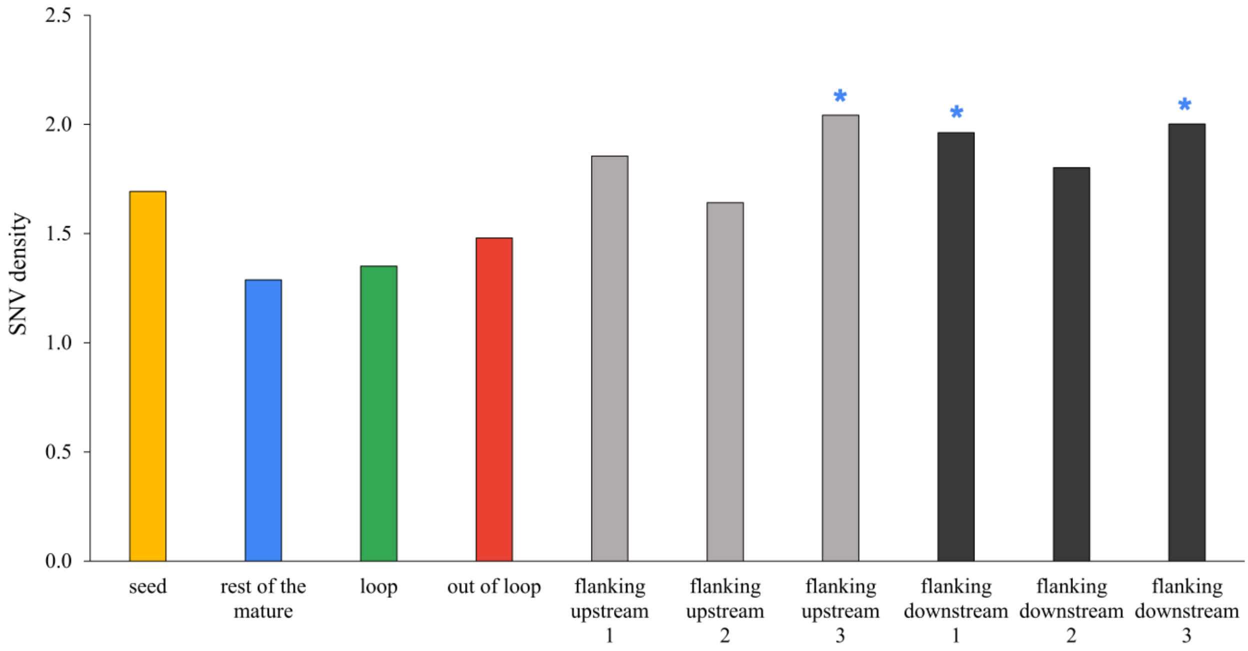

c) **Variants with AF > 1%**

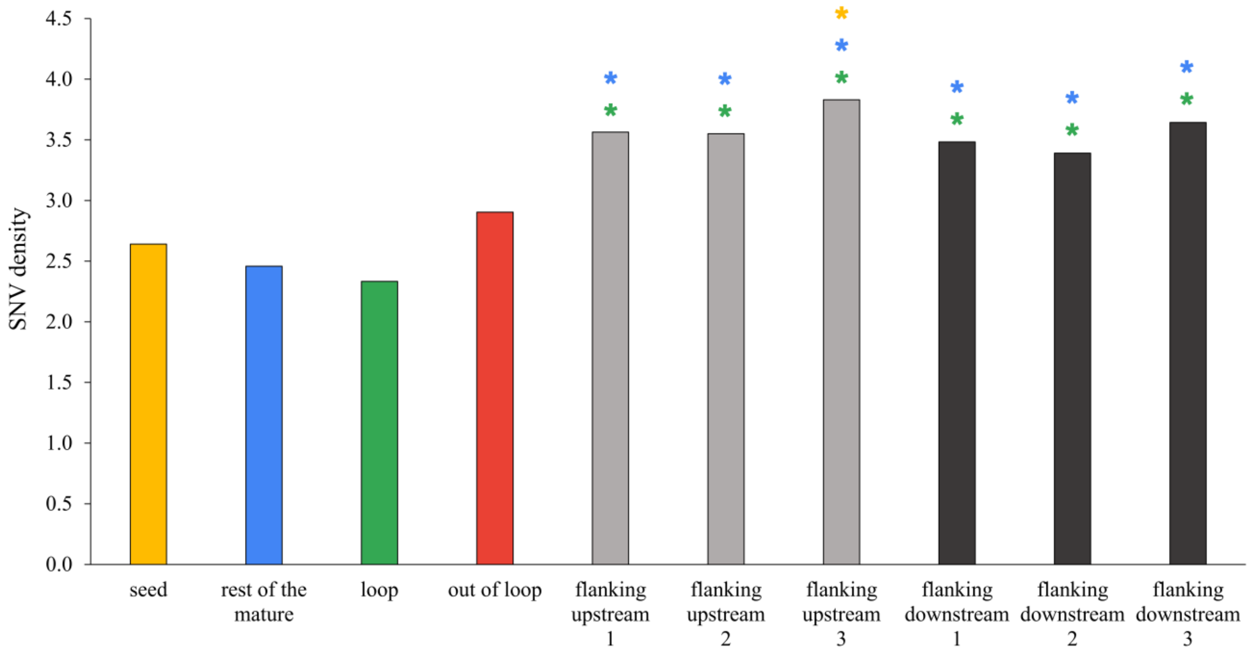

Supp. Figure S4.

Relationship among SNVs density, MiRLog average scores and phenotypes associated. (a) Spearman correlation for miRNAs associated with a least one phenotype in HMDD among SNVs density, number of phenotypes described, and MiRLog average predicted score. (b) Spearman correlation between SNVs density and MiRLog score for miRNAs associated with at least one phenotype. (c) SNVs density distribution for miRNAs associated with a phenotype (light blue) and those not associated (orange). To evaluate statistical differences, we used Mann-Whitney test ($p < 0.05$). (d) Spearman correlation between SNVs density and MiRLog score for miRNAs not associated with a phenotype.

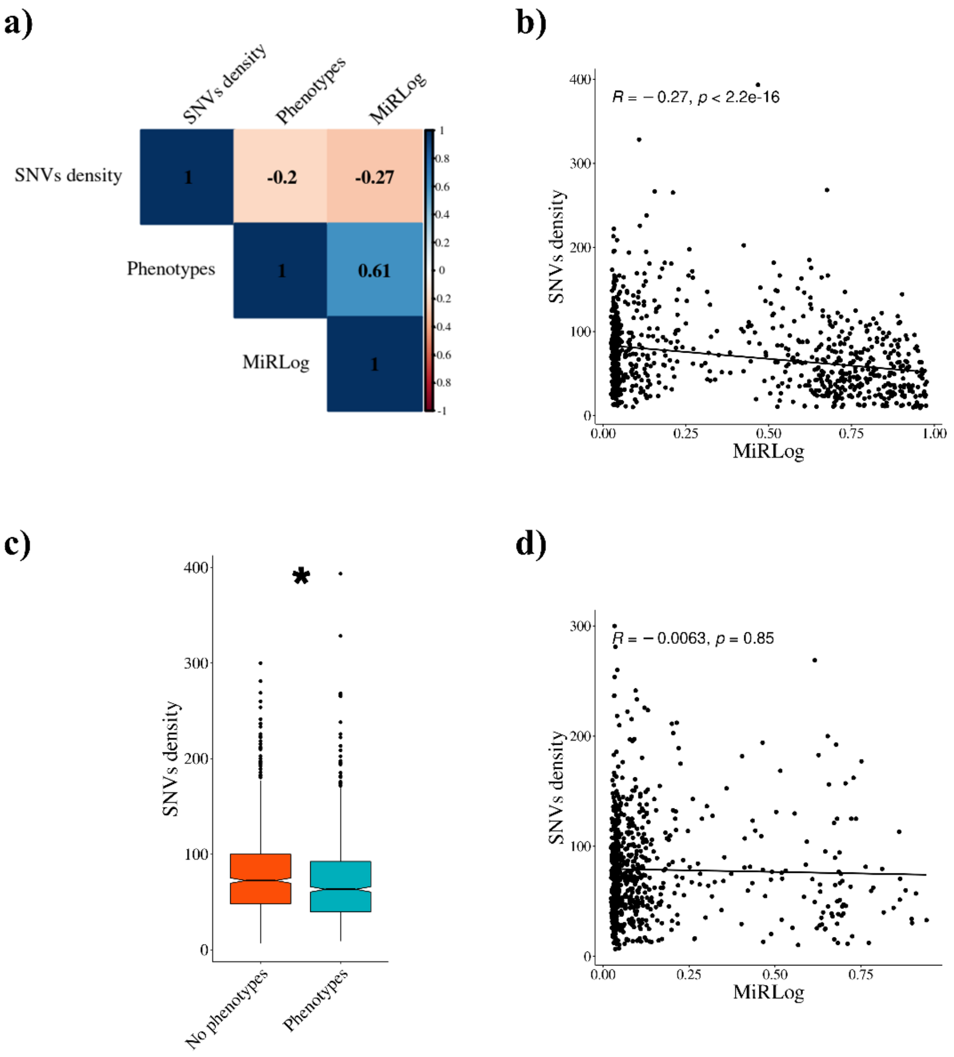

Supp. Table S3.

Hyperparameters explored in the grid search. To find the best hyperparameters used to develop MirLog classification pipeline we used a grid search. For each grid search hyperparameter we reported the algorithm and class object to which the parameter refers to, and the values tested. The best hyperparameters resulting from the grid search process are highlighted.

**Algorithm**	**Class object**	**Parameter**	**Values**									
Logistic regressor	sklearn.linear_model.LogisticRegression	C	0.01	0.04	0.09	0.16	0.25	0.36	0.49	**0.64**	0.82	1.00
Bagging Classifier	imblearn.ensemble.BalancedBaggingClassifier	max_features	0.3	0.4	0.5	0.6	0.7	0.8	**0.9**			
		sampling_strategy	**0.5**	0.6	0.7	0.8	0.9					

Supp. Table S4.
Full performance details of MiRLog.

	mean	median	std	min	max
**Accuracy**	0.96	0.95	0.04	0.85	1
**Balanced accuracy**	0.91	0.97	0.15	0.47	1
**Sensitivity**	0.84	1	0.29	0	1
**Specificity**	0.97	1	0.04	0.89	1
**Precision**	0.8	1	0.28	0	1
**Average precision**	0.91	1	0.15	0.42	1
**F1**	0.79	0.8	0.26	0	1

Supp. Table S5.

Functional annotation provided by dbmiR. We reported functional annotations available in dbmiR, indicating if it refers to miRNA or variant, the source, and its related link, when available. For each functional annotation we provid a category, as reported in Figure 2.

**Category**	**Functional annotation**	**miRNA/ variant**	**Source**	**Link**
variant deleteriousness	CADD	variant	CADD v1.4	https://cadd.gs.washington.edu/
variant deleteriousness	DANN	variant	DANN	https://cbcl.ics.uci.edu/public_data/DANN/
variant deleteriousness	ReMM	variant	ReMM 0.3.1	https://charite.github.io/software-remm-score.html
variant deleteriousness	LINSIGHT	variant	LINSIGHT	https://github.com/CshlSiepelLab/LINSIGHT
variant deleteriousness	ncER	variant	ncER	https://github.com/TelentiLab/ncER_datasets
variant deleteriousness	FATHMM-XF	variant	FATHMM-XF	http://fathmm.biocompute.org.uk/fathmm-xf/
variant deleteriousness	Eigen-PC	variant	regBase	https://github.com/mulinlab/regBase
variant deleteriousness	Funseq	variant	Funseq2.1.6	http://funseq2.gersteinlab.org/
variant deleteriousness	phyloP	variant	phyloP	https://genome.ucsc.edu/
variant deleteriousness	phastCons	variant	phastCons	https://genome.ucsc.edu/
variant deleteriousness	MiRLog	variant	implemented in this work	.
variant deleteriousness	PolymiRTS	variant	PolymiRTS-3.0	http://compbio.uthsc.edu/miRSNP/
variant effect on secondary structure	miRVaS	variant	miRVas	http://mirvas.bioinf.be/
variant identifier and frequency	variant identifier	variant	dbSNP152	https://www.ncbi.nlm.nih.gov/snp/
variant identifier and frequency	variant frequency	variant	gnomAD v2.1	https://gnomad.broadinstitute.org/
miRNA and variant localization	variants localization on miRNA domain and subdomain	variant	Manually curated	.

miRNA and variant localization	variant position on miRNA (HGVS nomenclature)	variant	Manually curated	.
miRNA and variant localization	MiRNA genomic localization	miRNA	Manually curated	.
miRNA and variant localization	miRNA known cluster	miRNA	Manually curated	.
miRNA and variant localization	miRNA predicted cluster	miRNA	Manually curated	.
miRNA and variant association with diseases	Known Mendelian disease-causing variant	variant	Literature retrieved	.
miRNA and variant association with diseases	ClinVar	variant	ClinVar (March 2019)	https://www.ncbi.nlm.nih.gov/clinvar/
miRNA and variant association with diseases	HGMD	variant	VEP 100	https://www.ensembl.org/info/docs/tools/vep/index.html
miRNA and variant association with diseases	COSMIC	variant	COSMICv89	https://cancer.sanger.ac.uk/cosmic
miRNA and variant association with diseases	Known Mendelian disease-causing miRNA	miRNA	Literature retrieved	.
miRNA association with phenotypes	HMDD	miRNA	HMDD 3.2	https://www.cuilab.cn/hmdd
miRNA association with phenotypes	PhenomiR	miRNA	PhenomiR 2.0	http://mips.helmholtz-muenchen.de/phenomir
miRNA association with phenotypes	HPO	miRNA	HPO	https://hpo.jax.org/app/
miRNA association with phenotypes	Monarch_Initiative	miRNA	Monarch Initiative	https://monarchinitiative.org/
miRNA target	TransmiR	miRNA	TransmiR 2.0	http://www.cuilab.cn/transmir
miRNA target	DIANA-TarBase	miRNA	DIANA-TarBase v7.0	http://diana.imis.athena-innovation.gr/DianaTools/
miRNA target	miRTarBase	miRNA	miRTarBase 7.0	http://mirtarbase.cuhk.edu.cn/php/index.php
miRNA target	TargetScan	miRNA	TargetScan 7.2	http://www.targetscan.org/vert_72/

miRNA expression	miRmine	miRNA	miRmine	https://guanfiles.dcmb.med.umich.edu/mirmine/
miRNA conservation	miRNA highly intolerant regions	miRNA	Orion	https://github.com/igm-team/orion-public
miRNA conservation	miRNA conserved regions	miRNA	CDTS	http://www.hli-opendata.com/noncoding/
miRNA nomenclature	miRNA nomenclature	miRNA	miRBase v20	http://www.mirbase.org/
miRNA nomenclature	miRNA gene symbol	miRNA	HGNC	https://www.genenames.org/
miRNA nomenclature	miRNA high confidence	miRNA	miRBase v20	http://www.mirbase.org/

Supp. Table S6.

Transitions and transversions observed in miRNAs and genome in 15,708 gnomAD WGS. We compared nucleotide changes (i.e., transitions and transversions) observed in miRNAs with those observed in the rest of the genome. Transversions indicated with a "*" were statistically lower in miRNAs than in the rest of the genome, while those indicated with a "†" were statistically lower in the rest of the genome compared to miRNAs. To evaluate statistical differences, we used Chi-square test ($p < 0.05$).

	**miRNA**		**genome**	
**Transitions**	**N° variants**	**Percentage**	**N° variants**	**Percentage**
**A/T > G/C**	3714	33.73%	66884976	32.78%
**G/C > A/T**	3663	33.27%	66761916	32.72%
**Transversions**	**N° variants**	**Percentage**	**N° variants**	**Percentage**
**A/T > C/G ***	920	8.36%	18201504	8.92%
**A/T > T/A***	535	4.86%	14761311	7.23%
**G/C > C/G†**	1296	11.77%	19296598	9.46%
**G/C > T/A***	882	8.01%	18146187	8.89%
**Total**	**N° variants**	**Percentage**	**N° variants**	**Percentage**
**Transitions**	7377	67.00%	133646892	65.49%
**Transversions***	3633	32.99%	70405600	34.50%
